# Chemerin Enhances Migration and Invasion of OC Cells via CMKLR1/RhoA/ROCK-Mediated EMT

**DOI:** 10.1155/2024/7957018

**Published:** 2024-07-26

**Authors:** Xiaojing Sun, Yi Guo

**Affiliations:** First Hospital of China Medical University Department of Obstetrics and Gynecology, Shenyang 110001, Liaoning, China

## Abstract

Chemerin is a newly described adipokine with significant effects on obesity, metabolic disorders, and immune trafficking. Recently, chemerin has gained prominence for its potential roles in cancer and tumorigenesis with pro- or antitumor effects. To date, most referenced multifunctions of chemerin are attributed to the chemokine-like receptor 1 (CMKLR1), distributing broadly in many tissues. This study investigates the *in vitro* roles of chemerin treatment on migration and invasion of ovarian carcinoma cells (OVCAR-3 and SK-OV-3) and potential underlying mechanisms. Herein, exogenous chemerin treatment promotes growth and invasion of SK-OV-3 cells but has no significant effects on OVCAR-3 cells. SK-OV-3 cells undergo morphological elongation characterized by epithelial-to-mesenchymal transition (EMT) and Ras homologous genome members A (RhoA)/Rho protein-related curl spiral kinase-1 (ROCK1) activation. Furthermore, chemerin-enhanced invasion and EMT of SK-OV-3 cells are effectively blocked by C3 transferase (C3T) and Y27632 and RhoA and ROCK1 inhibitor, respectively. More importantly, RhoA/ROCK1-EMT-mediated SK-OV-3 cell invasion is orchestrated by CMKLR1 upregulation after chemerin treatment (50 ng/mL). The silencing of CMKLR1 significantly (*P* < 0.0001) reverses the chemerin-enhanced invasion, EMT, and RhoA/ROCK1 activation of SK-OV-3 cells. Our study indicates that chemerin promotes invasion of OC cells via CMKLR1-RhoA/ROCK1-mediated EMT, offering a novel potential target for metastasis of OC.

## 1. Introduction

As a typical gynecological malignant tumor, ovarian cancer (OC) accounts for 4% of all female tumors [1, 2]. Over the past decade, occurrence of OC has increased about six times, and there is increasing incidence at younger ages [3, 4]. Moreover, OC is especially difficult to discover during the early stage due to the lack of diagnostic sign or symptoms and effective clinical method of screening [5]. Most patients will relapse within two years and the mortality rate of OC is highest among various gynecological tumors [6, 7]. The poor prognosis and high rate of recurrence are associated with the aggressive metastasis of OC cells into the abdominal cavity at the later stage [8]. About 85∼90% of OC are epithelial tumors, and most patients have distant peritoneal metastasis when diagnosed [9]. Therefore, more research studies are urgently needed to reveal the molecular mechanisms underlying metastasis of tumor cells and explore the potential interventions to improve the survival rate of patients.

Clinical studies show that omentum, a large fat pad, is the main site for subclinical metastasis of OC and nearly 80% of patients with ovarian carcinoma appear to have omental metastasis [10]. The omentum consists of adipocytes, functioning as an endocrine organ for releasing various adipokines, and energy storage site for lipid deposition [11]. It is interesting that both primary and recurrent advanced staged OC preferentially metastasize to adipose tissue, which indicates the importance of microenvironment of adipose tissue to the metastasis cascade of OC [12, 13].

Chemerin, a relatively newly discovered adipokine, is mainly secreted from adipose tissue with diverse biological functions, involved in the regulation of lipid synthesis, insulin resistance, and leukocyte chemoattractant [14–17]. In adipose tissue, it is found to regulate adipogenesis, lipolysis, and other metabolic activities [18]. Interestingly, a number of recent studies show the role of chemerin in different cancers with pro- and antitumor effects depending on context [19, 20]. It is found that OC patients have high levels of chemerin in their ascites, which is higher than serum levels [21, 22]. In addition, OC tissues contain higher levels of chemerin than those in adjacent tissues [22]. Some epithelial ovarian cells can synthesize and secrete chemerin, such as SK-OV-3 [23, 24], indicating that chemerin might largely exert its biological effects on ovarian cells. It has been reported that chemerin promotes growth and migration of HO8910 OC cells via PD-L1 upregulation [22]. An *in vitro* study confirms the proapoptotic role of chemerin on granulosa cells [25]. However, it has also been reported that chemerin *in vitro* promotes or does not affect the growth of ovarian cells, such as OVCAR-3 [24, 26]. These studies indicate that although chemerin has close association with OC development, research studies on its biological effects are limited.

Chemerin plays its role mainly by activating CMKLR1 receptor that is highly expressed in OC cells [24]. Chemerin induces metastasis of gastric cancer cells via CMKLR1 and CPR1/TIMPs pathways [27]. But it is unknown whether chemerin alters the metastasis of OC cells through CMKLR1. The novel finding shows that chemerin induces the activation of transcriptional regulator serum-response factor (SRF) via the Ras homologous genome members A/Rho protein-related curl spiral kinase-1 (RhoA/ROCK1) pathway mediated by binding with CMKLR1 [28]. The RhoA/ROCK pathway is critical for metastasis of tumor cells via stimulating the phosphorylation of myosin light chain (MLC), leading to the assembly of actin [29]. RhoA/ROCK activation also induces epithelial-to-mesenchymal transition (EMT) that exerts a vital role in tumor cell metastasis, especially for OC cells [30, 31]. However, whether chemerin regulates the metastasis of OC cells via the CMKLR1/RhoA/ROCK1 pathway has not been investigated. Herein, we explored the potential functions of exogenous chemerin on biological activities of OC cells, which reveal a previously undocumented role of CMKLR1/RhoA/ROCK1-mediated EMT for mechanical cues in OC metastasis.

## 2. Materials and Methods

### 2.1. Reagents

Recombinant human chemerin proteins were obtained from R&D system (Minneapolis, MN, USA, Cat. no. 2324-CM-025). Primary antibodies against CMKLR1 (ab64881) and vimentin (ab92547) were obtained from Abcam (Cambridge, UK). E-cadherin (14472), N-cadherin (14215), RhoA (2117), ROCK1 (28999), p-MLC2 (3675), MLC (8505), and ZEB1 (3396) were obtained from Cell Signaling Technology (CST, Danvers, USA). The inhibitor of RhoA, C3 transferase (C3T), was brought from Cytoskeleton (CT03, Denver, USA) and the ROCK1 inhibitor, Y27632, was obtained from Cayma Chemical (129830-38-2, Michigan, USA).

### 2.2. Cell Culture and Chemerin Treatment

OVCAR-3 and SK-OV-3, two human OC cell lines, were obtained from American Type Culture Collection (ATCC, Manassas, VA) and were incubated within RPMI 1640 medium (Gibco, NY, USA) containing 100 U/ml penicillin, 100 *μ*g/ml streptomycin, and 10% fetal bovine serum (Gibco, NY, USA) at 37°C and 5% CO_2_ incubator. For cell growth detection, both cells were cultured on 96-well plates and were treated with different doses of chemerin (0, 25, 50, and 100 ng/ml) for 24, 48, and 72 h. For western blot analysis, both cells were cultured on 6-well plates and were stimulated with chemerin (50 ng/ml) for 24 h. SK-OV-3 cells were pretreated with the RhoA inhibitor, C3T (1.5 *μ*g/ml), or the ROCK1 inhibitor, Y27632 (30 *μ*M), for 2 h and then were treated with chemerin (50 ng/ml) for another 24 h.

### 2.3. Silencing of CMKLR1

The siRNAs for CMKLR1 (two silencing sequences) and negative control (NC) were synthesized by GenePharma (Shanghai, China). SK-OV-3 cells were cultured into a 6-well plate and transfected with indicated CMKLR1 siRNA using Lipofectamine 8000 (Beyotime, Shanghai, China) according to manufacturer's instruction for 48 h. Then, the silenced cells were treated with chemerin (50 ng/ml) for another 24 h. The silencing sequences are presented in the following:

  si-CMKLR1#1: AAUAAUCAGGGUAUUCAUCAC
  si-CMKLR1#2: ACAUGUUGUGGAUGAGAAGAA

### 2.4. Western Blot Analysis

Cells were cultured on 6-well plates and treated with chemerin (50 ng/mL) for 24 h. After treatment, supernatants were collected and lysed with a mixture of protease inhibitor RIPA lysis buffer (Beyotime, Haimen, China) for 40 min at 4°C. After centrifugation at 13,780 g for 10 min, the protein solution was transferred into a new tube. The BCA kit (WLA004B, Wanleibio, China) was used to detect the protein content according to manufacturer's instruction, then it was denatured by 5x loading buffer (Solarbio, Beijing, China) at 100°C for 5 min. The 10–12% SDS-PAGE was prepared to separate the target proteins, and the proteins were transferred into PVDF membranes. After immersing into blocking solution for 2 h, the membranes containing targeted proteins were incubated with primary antibodies at 4°C overnight. Then, the membranes were treated with horseradish-peroxidase-conjugated secondary Abs after washing with PBS for three times. The proteins were visualized after incubation with enhanced chemiluminescence (ECL) (Thermo Fisher Scientific, Rockford, IL, USA) using ChemiDoc MP chemiluminescence gel imaging system (Bio-Rad, California, USA).

### 2.5. RT-qPCR Analysis

Cells were treated with chemerin (50 ng/mL) or silenced with CMKLR1 si-RNAs or RhoA/ROCK1 inhibitors for indicated time. RNAiso plus reagent (Takara, Tokyo, Japan) was applied to extract the total RNA following the manufacturer's instructions. Then, RNA (1 *μ*g) was used for reverse transcription using PrimeScriptTMRT Master Mix (Takara). The relative level of target mRNA was confirmed by comparing with *β-actin* and GAPDH mRNA according to the following conditions: initial denaturation at 95°C for 15 min, followed by 40 cycles of 10 s denaturation at 95°C, 5 s annealing at 60°C, and 12 s extension at 72°C. The relative mRNA levels were the mean 2^−(∆∆Ct)^ value compared with *β*-actin and GAPDH. They were used as internal reference genes due to their highly conserved sequence and high mRNA expression in OC cells, which contributes to normalize the other gene expression. The Primer-BLAST software (NCBI) was used to designed primers for these genes. Primer sequences, annealing temperature, and amplification length are shown in Table 1.

### 2.6. Cell Proliferation

After seeding OVCAR-3 and SK-OV-3 cells (8 × 10^4^/ml) on 96 wells, they were stimulated with chemerin (25, 50, and 100 ng/mL) for indicated times (24, 48, and 72 h). Then, cell proliferation was detected using Cell Counting Kit 8 (CCK-8) (Beyotime, Haimen, China) according to manufacturer's instruction. The cell numbers were presented by the OD value at 450 nm.

### 2.7. Cell Migration

Cells (10 × 10^4^/ml) were cultured on 24-well dishes for 90% confluence and then were vertically scratched from top to bottom using a tip. After washing three times with PBS, cells were incubated with different doses of chemerin (25, 50, and 100 ng/mL) for 24 h in serum-free medium. The cell scratches were captured at 0 and 24 h after chemerin treatment. The cells migrated onto the scratched-off region were captured using a phase contrast microscope, and the scratched areas were quantified using Image Pro Plus 6.0 software. The migration ratio was calculated as follows:

Migration ratio (%) = (premigratory scratched open area − postmigratory open area)/(premigratory scratched open area) × 100%.

### 2.8. Transwell Analysis

SK-OV-3 and OVCAR-3 cells or CMKLR1-silenced SK-OV-3 cells were treated with chemerin (50 ng/ml) for 24 h. Then, cells were collected and recultured (2 × 10^4^ cells/200 uL) on the upper insert coated with Matrigel (BD Bioscience, san Joes, USA) of a transwell plates (Corning, NY, USA) with an 8 *μ*m pore size filters for 4 h with the serum-free medium. Cells transferred into the lower chambers were determined and stained using 0.1% crystal violet for 30 min. Invading cells were counted using Image J software.

### 2.9. Statistical Analysis

At least three independent experiments were applied for data collection. All data were calculated and analyzed with GraphPad Prime software. One-way ANOVA was applied to examine the comparison among the groups. *P* < 0.05 was described as the statistical significance.

## 3. Results

### 3.1. Differentiated Regulation of Exogenous Chemerin on Cell Growth in OVCAR-3 and SK-OV-3 Cells

**Figure 1:**
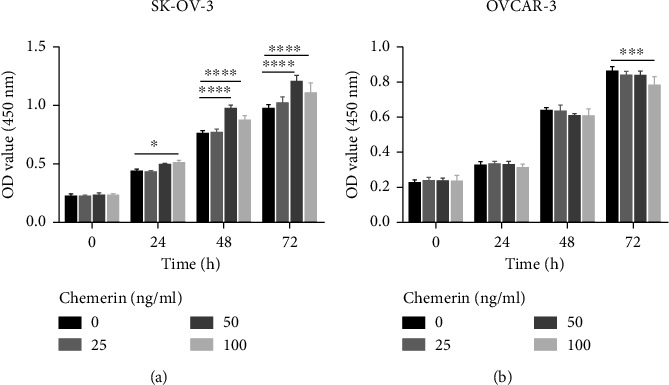
Effect of chemerin on cell growth in SK-OV-3 and OVCAR-3 cells. (a-b) SK-OV-3 and OVCAR-3 cells were treated with chemerin (25, 50, and 100 mg/mL) for 24, 48, and 72 h. The cell growth was presented as the OD value at 450 nm. Significance was determined by one-way ANOVA analysis with Tukey's post hoc test. ^*∗*^*P* < 0.05; ^*∗∗∗*^*P* < 0.001; ^*∗∗∗∗*^*P* < 0.0001. Data are plotted as the mean ± SD.

The results of cell proliferation showed that lower dose of chemerin (25 ng/mL) did not influence the cell growth at any time points, but higher concentrations of chemerin (50 and 100 ng/mL) significantly promoted the proliferation of SK-OV-3 cells after 48 h and 72 h incubation (*P* < 0.0001). Furthermore, a more significant enhanced-growth role was observed after chemerin treatment at 50 ng/mL (Figure 1(a)). However, the different doses of chemerin treatment had no significant effect on the proliferation of OVCAR-3 cells at 24 and 48 h. Only higher concentration of chemerin (100 ng/mL) had an inhibitory role in cell growth at 72 h (Figure 1(b)).

### 3.2. Chemerin Enhances Migration and Invasion of SK-OV-3 Cells

**Figure 2:**
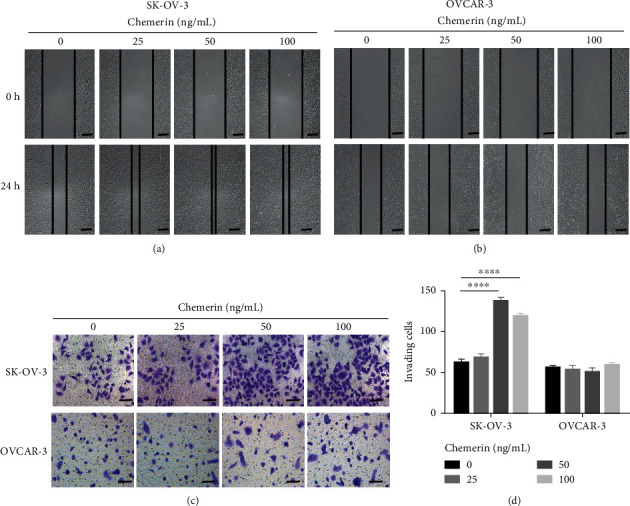
Migration and invasion of SK-OV-3 and OVCAR-3 cells treated with chemerin. (a-b) SK-OV-3 and OVCAR-3 cells were scratched and treated with chemerin (25, 50, and 100 mg/mL). The cell migration was observed after 24 h scratch. Scale bar = 200 *μ*m. (c) The invasion of SK-OV-3 and OVCAR-3 cells treated with chemerin (25, 50, and 100 mg/mL) was measured using transwell. Scale bar = 100 *μ*m. (d) Quantification of invading cell numbers in the transwell experiment. Significance was determined by one-way ANOVA analysis with Tukey's post hoc test. ^*∗∗∗∗*^*P* < 0.0001. Data are plotted as the mean ± SD.

The influence of different concentrations of chemerin on cell movement in scratch and transwell processes was explored, and it was found that different doses of chemerin significantly (*P* < 0.0001) accelerated the healing of SK-OV-3 cells after 24 h scratch, indicating the enhanced cell migration after chemerin treatment (25, 50 and 100 ng/mL) (Figure 2(a)). Moreover, chemerin has no obvious influence on the migration of OVCAR-3 cells at any concentration (Figure 2(b)). Consistently, the results of transwell also indicated the enhanced invasion of SK-OV-3 cells after chemerin treatment at different doses (Figure 2(c)). Moreover, chemerin at 50 ng/mL presented a more significant (*P* < 0.0001) enhancement on migration and invasion of SK-OV-3 cells (Figure 2(c)). However, chemerin did not affect invasion of OVCAR-3 cells at different doses (Figure 2(d)). These data suggest that chemerin might be inclined to regulate biological activities of the more-metastatic OC cells.

### 3.3. Chemerin Activates the CMKLR1/RhoA/ROCK1 Pathway and EMT

**Figure 3:**
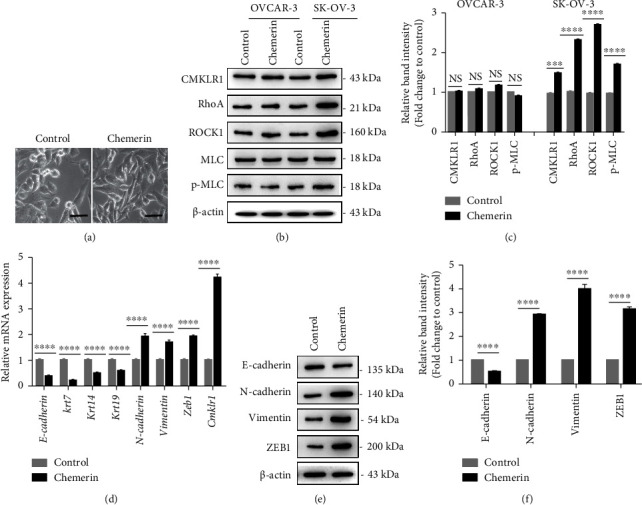
Chemerin promotes CMKLR1-RhoA/ROCK and EMT in SK-OV-3 cells. (a) The phase contrast image of SK-OV-3 cells after 24 h treatment with chemerin (50 ng/mL). Scale bar = 50 *μ*m. (b) Western blot analysis of CMKLR1, RhoA, ROCK1, and MLC protein expressions and the level of p-MLC in SK-OV-3 and OVCAR-3 cells treated with chemerin (50 ng/mL) for 24 h. *β*-actin is used as a loading control. (c) Relative quantification of CMKLR1, RhoA, and ROCK1 expressions to *β*-actin expression, and the phosphorylation level MLC relative to MLC in B. (d) The mRNA levels of genes involving Cmklr1, Krt7, 14, 19, E-cadherin, N-cadherin, vimentin, and Zeb1. *β*-actin and GAPDH were used as internal reference genes. (e) Western blot analysis of N-cadherin, E-cadherin, vimentin, and ZEB1 expressions in SK-OV-3 cells treated with chemerin (50 ng/mL) for 24 h. *β*-actin is used as a loading control. (f) Quantification of N-cadherin, E-cadherin, vimentin, and ZEB1 expressions in E. ^*∗∗∗*^*P* < 0.001; ^*∗∗∗∗*^*P* < 0.0001. Data are plotted as the mean ± SD.

CMKLR1 is the main binding site of chemerin in the regulation of cell behaviors; we then investigated the expression of CMKLR1 and potential downstream signaling pathway in SK-OV-3 and OVCAR-3 cells treated with chemerin (50 ng/mL) for 24 h. The images showed that chemerin (50 ng/mL) treatment increased the cell polarity with morphological elongation of cells, the EMT characteristic, in SK-OV-3 cells (Figure 3(a)). Western blot analysis showed that chemerin addition evidently increased the expression of CMKLR1 in SK-OV-3 cells (Figures 3(b) and 3(c)). Our results showed that chemerin treatment also promoted the expression of RhoA, ROCK1, and the level of phosphorylation of MLC in SK-OV-3 cells, which attributed to cytoskeleton organization and migration/invasion, but it has no significant effect on protein expressions in OVCAR-3 cells (Figures 3(b) and 3(c)). Therefore, only SK-OV-3 cells were used for the subsequent experiments. The RT-PCR analysis indicated that chemerin treatment increase the expression of Cmklr1 and the mesenchymal indicators, N-cadherin, vimentin, and Zeb1 while decreased the expression of epithelial markers, such as E-cadherin, Krt7, 19, and 14 in SK-OV-3 cells (Figure 3(d)). Furthermore, chemerin treatment downregulated E-cadherin and upregulated vimentin, N-cadherin, and ZEB1 expressions in SK-OV-3 cells (Figures 3(e) and 3(f)). These results indicated CMKLR1-RhoA/ROCK1 and EMT might be associated with chemerin-enhanced migration and invasion of SK-OV-3 cells.

### 3.4. The Silencing of CMKLR1 Reverses the Chemerin-Enhanced Invasion of SK-OV-3 Cells

**Figure 4:**
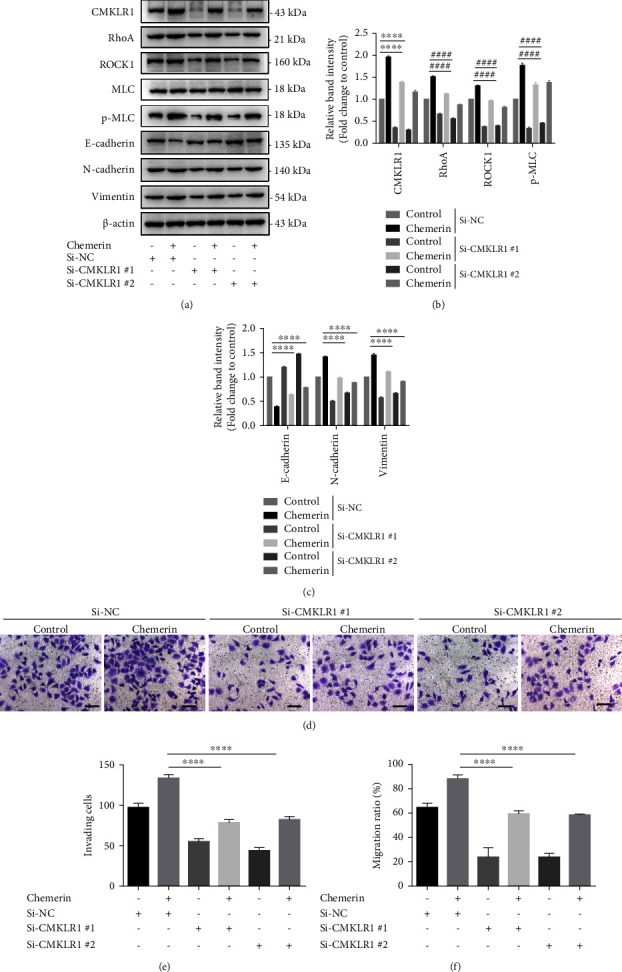
The silencing of CMKLR1 reverses chemerin-enhanced invasion of SK-OV-3 cells and RhoA/ROCK/EMT activation. (a) Western-blot analysis of CMKLR1, RhoA, ROCK1, MLC, p-MLC, N-cadherin, E-cadherin, and vimentin in SK-OV-3 cells silenced with siRNAs targeted with CMKLR1. (b) Relative quantification of CMKLR1, RhoA, and ROCK1 expressions to *β*-actin and the phosphorylation level of MLC relative to MLC in A. (c) Quantification of N-cadherin, E-cadherin, and vimentin expressions relative to *β*-actin expression in A. (d) The invasion of SK-OV-3 cells silenced with CMKLR1 siRNA was measured using transwell assay. (e) The invasion of SK-OV-3 and OVCAR-3 cells silenced with siRNAs targeted with CMKLR1 was measured using transwell. Scale bar = 100 *μ*m. (f) Quantification of invading cell numbers in the transwell experiment. Significance was determined by one-way ANOVA analysis with Tukey's post hoc test. ^*∗∗∗∗*,####^*P* < 0.0001. Data are plotted as the mean ± SD.

To further verify that whether CMKLR1 is necessary for the effects of chemerin on RhoA/ROCK1-mediated EMT and enhanced movement of SK-OV-3 cells, the cells were transfected with CMKLR1 siRNAs before chemerin treatment. Western blot analysis showed the significantly (*P* < 0.0001) decreased CMKLR1 expression after transfection of siRNA #1 and #2 (Figures 4(a) and 4(b)). The chemerin-enhanced expressions of RhoA, ROCK1, and the phosphorylation level of MLC were also reversed by the silencing of CMKLR1 (Figures 4(a) and 4(b)). Consistently, the downregulation of CMKLR1 also inhibited EMT process with increased E-cadherin expression as well as decreased N-cadherin and vimentin expressions in SK-OV-3 cells treated with chemerin (Figures 4(a) and 4(c)). Moreover, the results of transwell and cell scratch also indicated that the silencing of CMKLR1 significantly (*P* < 0.0001) reduced invasion and migration of SK-OV-3 cells with less invading cells than chemerin treatment alone (Figure 4(d), 4(e), and 4(f)). These data proved that CMKLR1 is required for chemerin-enhanced invasion as well as the activation of RhoA/ROCK1 and EMT in SK-OV-3 cells.

### 3.5. The Inhibition of RhoA/ROCK1 Pathway Suppresses the Chemerin-Induced EMT

**Figure 5:**
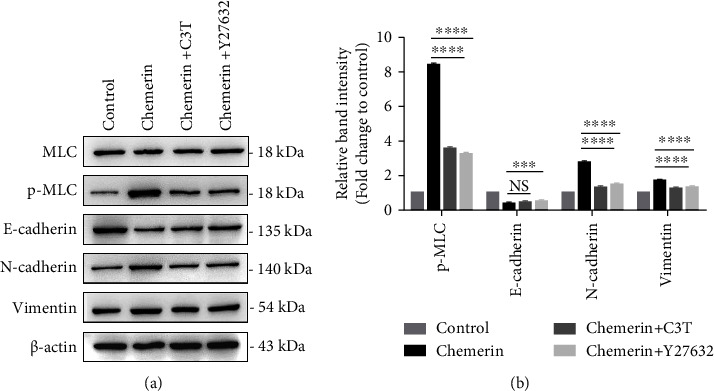
RhoA/ROCK1 is necessary for chemerin-promoted EMT. (a) SK-OV-3 cells were pretreated with RhoA inhibitor, C3T (1.5 *μ*g/ml) or ROCK1 inhibitor, Y27632 (30 *μ*M) for 1 h, and then were treated with chemerin (50 ng/mL). Western blot analysis of MLC, p-MLC, N-cadherin, E-cadherin, and vimentin expressions. (b) Relative quantification of p-MLC to MLC, and N-cadherin, E-cadherin, and vimentin to *β*-actin expression in A. Significance was determined by one-way ANOVA analysis with Tukey's post hoc test. ^*∗∗∗*^*P* < 0.001; ^*∗∗∗∗*^*P* < 0.0001; NS, not significant. Data are plotted as the mean ± SD.

To elucidate whether chemerin enhances EMT of SK-OV-3 cells in a RhoA/ROCK1 dependent manner, cells were pretreated with either C3T (1.5 *μ*g/ml, RhoA inhibitor), Y27632 (30 *μ*M, ROCK1 inhibitor), or vehicle control. The results showed that RhoA/ROCK1 inhibition decreased the levels of MLC phosphorylation and hence verified the action of inhibitor (Figure 5(a)). The expression of EMT-related proteins was not activated by chemerin treatment in SK-OV-3 cells pretreated with C3T or Y27632. Chemerin decreased the expression of E-cadherin and increased expression of N-cadherin and vimentin; however, their expressions were reversed by C3T or Y27632 pretreatment (Figures 5(a) and 5(b)). Overall, these results revealed that chemerin promotes invasion of SK-OV-3 OC cells via targeting CMKLR1 that attributed to activation of RhoA/ROCK pathway-mediated EMT.

## 4. Discussion

The intention of study was to investigate the effect of chemerin on biological behaviors of OC cells and reveal the underlying specific mechanism. We identified that chemerin functioned as a promotion factor for SK-OV-3 cell proliferation and migration through CMKLR1/RhoA/ROCK-mediated EMT. The present study not only enriches the researches of chemerin in female malignant tumor development especially for omental metastasis but also reveals a novel mechanism of CMKLR1/RhoA/ROCK1-mediated EMT in invasion of metastasis of OC cells.

Increasing studies show that chemerin plays a dual effect on tumor development. Chemerin expression and CMKLR1 levels have been found to be upregulated in cases of cancer cells (glioblastoma, mesothelioma, and squamous cell carcinoma of the oral tongue), exacerbating tumor expansion and metastasis [32–35]. Conversely, chemerin seems to be a suppressive factor in some cases, involving acute myeloid leukemia, hepatocellular carcinoma, breast cancer, etc. [36–40]. However, the role of chemerin in ovarian cancer cells is largely elusive. Herein, we found that chemerin has a paradoxical effect on proliferation of OVCAR-3 and SK-OV-3 cells. It was observed that chemerin (25, 50, and 100 ng/mL) treatment enhanced SK-OV-3 cell growth, but high dose of chemerin (100 ng/mL) inhibited OVCAR-3 cell growth. We infer that doses, exposure time of chemerin, or difference in type of ovarian cancer cells might account for its variation in biological function. It was reported that chemerin did not influence the growth of ovarian noncancer cells (HOSEpiC) and OVCAR-3 and COV434 cells at doses from 3-300 ng/mL after 48 h [24]. It is consistent with our results that chemerin did not affect the growth of OVCAR-3 cells after treatment for 24 and 48 h. Gao Chenxi et al. reported that chemerin addition apparently promoted cell proliferation in HO8910 OC cells but had no impact on HO8910PM cells [22]. These data suggested that chemerin might primarily exert a distinct role in different types of OC cells. Furthermore, the underlying mechanism of chemerin on OC development is largely unclear.

We also found that chemerin had a biphasic role in cell movement (migration and invasion) in SK-OV-3 and OVCAR-3 cell lines. This may be due to the difference in migration characteristics of two cells. It has been proven that SK-OV-3 is a type of high-metastatic cell line that is more sensitive to exogenous stimuli compared to the low-metastatic OVCAR-3 cells [41, 42]. A previous study has shown that the treatment of recombinant versican and hyaluronan (HA) resulted in invasion promotion in SK-OV-3 cells but had no effect on OVCAR-3 cells, which lack the HA receptor and CD44 [43]. The nitric oxide donors reduced the transmigration of SK-OV-3 cell by affecting metalloproteinase 2 (MMP2), which was not changed in OVCAR-3 cells [44]. Furthermore, compared with SK-OV-3 cells, there was no obvious morphology shift from epithelial phenotype to a more mesenchymal phenotype in OVCAR-3 cells after treatment of chemerin. Mechanistically, we found that chemerin treatment did not induce the activation of CMKLR1/RhoA/ROCK1-mediated EMT in OVCAR-3 cells, which might account for the difference in movement in two cell lines. Therefore, we speculated that the inactivation of CMKLR1/RhoA/ROCK1-mediated EMT might be the potential reason for the difference in cell movement in OVCAR-3 cells.

It is unclear that how chemerin regulates biological behaviors of OC cells. Lu Zhiyuan et al. showed that chemerin addition increased proliferation, migration, and invasion of oral squamous cells carcinoma (OSCC) cells with the upregulation of antioxidative activities and EMT [45]. It was demonstrated that vascular smooth muscle cell behaviors (growth and migration) were regulated by chemerin exogenous treatment via CMKLR1/lipocalin-2 axis that induced the activation of p38/MAPK and Wnt/*β*-catenin pathways [46]. It was also reported that chemerin suppressed metastasis of hepatocellular carcinoma cells through the CMKLR1-PTEN-AKT axis [47]. Our work revealed that the CMKLR1-RhoA/ROCK-EMT signaling pathway might be a novel pathway for the functions of chemerin on proliferation and invasion of OC cells. It is shown that the RhoA/ROCK1 pathway is a novel chemerin/CMKLR1 effector in mediating the activation of various transcription factors that is required for cell proliferation, migration, and invasion [48, 49]. Therefore, we concluded that the impact of chemerin on cell proliferation in OC cells might be related with RhoA/ROCK1 activation; however, the specific mechanism needs to be further established.

Taken together, this work clearly illustrated the functions of chemerin on cell proliferation, migration, and invasion in OC cell. We pointed out an undocumented mechanism involving the CMKLR1/RhoA/ROCK1-mediated EMT pathway that might be a prospective indicator for the therapies of high metastasis ovarian malignant cancers.

## Figures and Tables

**Table 1:** The information for primer sequences, annealing temperature, and amplification length.

Primer sequences	Annealing temperature (°C)	Amplification length (bp)	Accession number
*E-cadherin* forward, 5′-CGAGAGCTACACGTTCACGG-3′ *E-cadherin* reverse, 5′-GGGTGTCGAGGGAAAAATAGG-3′	55	119	AC099314.3

*Krt7* forward, 5′-TCCGCGAGGTCACCATTAAC-3′; *Krt7* reverse, 5′-GCTCTGTCAACTCCGTCTCAT-3′;	55	519	AC021066.37

*Krt14* forward, 5′-TGAGCCGCATTCTGAACGAG-3′; *Krt14* reverse, 5′-GATGACTGCGATCCAGAGGA-3′;	55	474	AC019349.28

*Krt19* forward, 5′-AACGGCGAGCTAGAGGTGA-3′; *Krt19* reverse, 5′-GGATGGTCGTGTAGTAGTGGC-3′;	55	91	AB045973.1

*Zeb1* forward, 5′-TTACACCTTTGCATACAGAACCC-3′; *Zeb1* reverse, 5′-TTTACGATTACACCCAGACTGC-3′;	55	100	AL117340.3

*Vimentin* forward, 5′-AGTCCACTGAGTACCGGAGAC-3′; *Vimentin* reverse, 5′-CATTTCACGCATCTGGCGTTC-3′;	55	98	AL133415.13

*N-cadherin* forward, 5′-TCAGGCGTCTGTAGAGGCTT-3′; *N-cadherin* reverse, 5′-ATGCACATCCTTCGATAAGACTG-3′;	55	94	AC006249.1

*CMKLR1* forward, 5′-CAGTTACGGTGATGAATACCCTG-3′ *CMKLR1* reverse, 5′-GACGATGCTGTAGACCACCAC-3′	55	115	AB065871.1

*β-actin* forward, 5′-CATGTACGTTGCTATCCAGGC-3′; *β-actin* reverse, 5′- CTCCTTAATGTCACGCACGAT-3′	55	250	AC006483.3

## Data Availability

The data used to support the findings are available from the corresponding author upon request.
